# Cutaneous leiomyosarcoma on the trunk: An unusual presentation with an aggressive course – Case report and review of literature

**DOI:** 10.11604/pamj.2018.31.190.16682

**Published:** 2018-11-19

**Authors:** Lina Hmida, Feryel Letaief, Raoudha Doghri, Khedija Meddeb, Khalil Mahjoubi, Amina Mokrani, Yosra Yahiaoui, Azza Gabsi, Nesrine Cheraiet, Henda Rais, Mouna Ayadi, Amel Mezlini

**Affiliations:** 1 Medical Oncology Department, Salah Azaiez Institute, Tunis, Tunisie; 2 Anatomic Pathology Department, Salah Azaiez Institute, Tunis, Tunisie; 3 Radiotherapy Department, Salah Azaiez Institute, Tunis, Tunisie

**Keywords:** Leiomyosarcoma cutaneous, chemotherapy, surgery

## Abstract

Primary cutaneous leiomyosarcoma (PCL) are soft-tissue sarcoma, arising in the dermis, with or without extension into the subcutis. They are thought to have an indolent course compared to their subcutaneous counterparts, they may recur but rarely metastases. We report the case of a patient with a PCL arising in the anterior trunk wall who developed pulmonary, bone and retroperitoneal metastases 6 years after wide surgical excision of the primary tumor.

## Introduction

Leiomyosarcoma (LMS) are soft-tissue sarcoma which arise from smooth muscles. They can involve either peripheral soft tissues sites also called superficial LMS or deep soft tissues sites. Superficial LMS are rare mesenchymal neoplasms accounting for about 7% of soft-tissue neoplasms and 0.04% of all cancers. They have two subdivisions, cutaneous and subcutaneous forms basis of their histogenesis and different clinical and prognostic implications. Primary cutaneous leiomyosarcoma (PCL) are the rarest subtype, arising in the dermis, with or without extension into the subcutis. They are thought to have an indolent course compared to their subcutaneous counterparts; they may recur but rarely metastases. We report the case of a patient with a PCL arising in the anterior trunk wall who developed pulmonary, bone and retroperitoneal metastases 6 years after wide surgical excision of the primary tumor.

## Patient and observation

A 30-year-old, otherwise healthy male presented to the outpatient department of dermatology in 2007 with a slow growing mass on his anterior chest wall. The swelling was present for more than 10 years without causing any complaint. During the year before presentation, the mass had progressively increased in size and became tender. No history of trauma, infection or radiation was present. The physical examination found an exophytic, reddish-brown swelling of 5cm with central ulceration of 2cm arising from the right pre-sternal region. A cutaneous biopsy was taken. It revealed a dermal fusocellular proliferation with pleomorphic and atypical nuclei but no evidence of mitosis. Hence, the diagnosis of atypical leiomyoma was rendered. Based on the above findings, the patient was referred to surgical department. Complete excision of the lesion was carried out through a periareolar incision. The distance of the tumor from the nearest resection margin was 1cm. Subcutaneous tissue and fascia of the pectoralis major was removed too.

**Figure 1:**
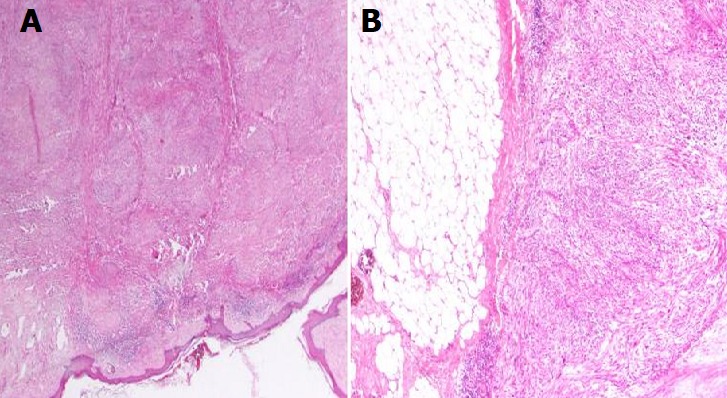
Histopathological examination of the excised skin nodule (10 fold magnification: A) shows fusocellular proliferation in the superficial and deep dermis; B) with high cellularity and focal extension to the hypodermis

**Figure 2:**
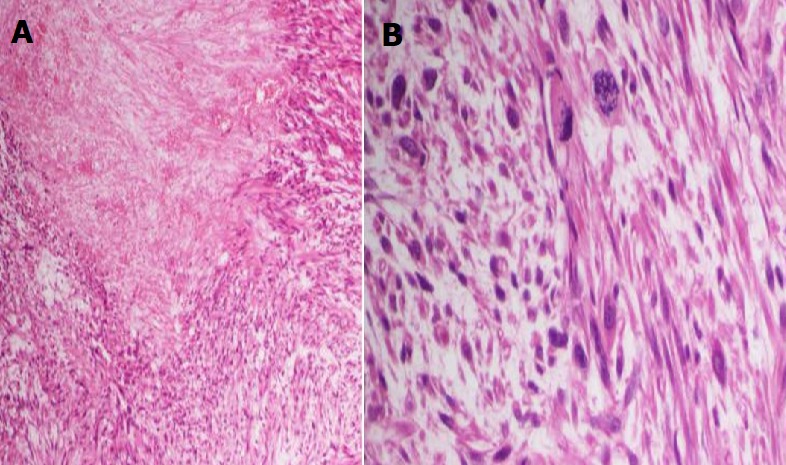
Histopathological examination of the excised skin nodule (100 fold magnification: A) shows numerous atypical mitotic figures; B) and large foci of tumoral necrosis

The excised skin nodule revealed a mass measuring around 4.5×4.5×2cm located in the dermis. Histopathological examination found a highly cellular mesenchymal proliferation especially in the deeper portions of the tumor. Numerous atypical mitotic figures were noted ranging from 4 per 10 high-power fields in the superficial portion to 16 per 10 high-power fields in the deeper portions. Eosinophilic cytoplasm with hyperchromatic and pleomorphic nuclei were seen too. The tumor showed large foci of tumoral necrosis (< 50%) and very limited extension to the hypodermis without vascular invasion (Figure 1, Figure 2). In immunohistochemistry (IHC), cells were positive for smooth muscle actin (SMA) and caldesmon but negative for CD34. All the margins were free. Hence, cutaneous leiomyosarcoma with limited extension to the subcutis was diagnosed. Adjuvant chemotherapy or radiation therapy was not considered at this point by the multidisciplinary oncology team.

After 3 years of regular follow-up, the patient was doing well with no clinical or radiological sign of recurrent or metastatic disease and then decided to abandon the routine follow up against medical advice. Six years postoperatively, he was admitted in our medical department of oncology with a six months history of a painless rapidly increasing mass of the left iliac fossa. There was no fever, transit disorder, vomiting or body weight loss. Physical examination discovered an 11cm mass of the left iliac fossa arising from the pelvis. The overlying skin was normal. No neurological deficits were detected. A keloid scar of 2cm was found on the right presternal region.

**Figure 3:**
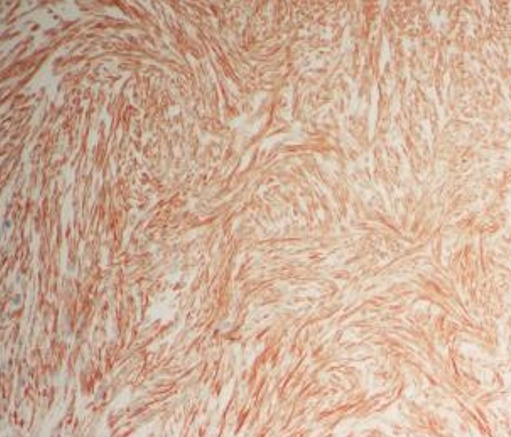
Immunohistochemistry (IHC) of the excised pelvic mass revealed that the lesion was caldesmon positive

Subsequent X-ray of the pelvis showed osteolytic lesions in the left iliac bone. Pelvis MRI depicted a tissular mass measuring 13cm x 13cm x 10 cm involving the psoas iliac and gluteal muscles until the iliac crest. A second tissular mass of 5 x 4 x 3cm was seen around the left ischio-pubic branch with hyperfixation in bone scintigraphy. Thoracoabdominopelvic computed tomography (TAP-CT) demonstrated bilateral lung metastases of 7mm to 45mm too. A biopsy of the pelvic mass was performed and led to diagnosis of leiomyosarcoma (Figure 3).

The case was discussed in a multidisciplinary consultation meeting indicating medical treatment be with doxorubicin and ifosfamide regimen. After 6 courses of chemotherapy, tumor response was compared according to the RECIST 1.1 criteria concluding to disease stabilization. A break from chemotherapy was allowed after discussion with the patient. During this period, he was followed up regularly and was evaluated by computed tomography scan every 3 months or when clinically indicated. The patient remained stable for almost 16 months until CT scans revealed significant disease progression. Then subsequent salvage second-line chemotherapy with high dose ifosfamide was given. Unfortunately, the patient showed rapid disease progression during the third course of treatment. Since performance status was maintained, he was eligible to receive third-line chemotherapy. Thus, disease stabilization in the following six months was achieved with gemcitabine monotherapy. Recently, patient’s performance status (PS) has deteriorated and further clinical progression was detected, so we began a 4^th^ line therapy with metronomic oral cyclophosphamid.

## Discussion

Primary Cutaneous Leiomyosarcomas (PCL) are highly-infrequent mesechymatal malignant neoplasm which are thought to arise from the arrector pili muscles in the dermis. They were described for the first time in 1959 by Montgomery and Winkleman [1]. Histogenesis remains unclear but physical trauma, radiation exposure and medical history of leiomyoma are suspected to be the most predisposing factor [1]. Association with burn and small pox scars was reported too [2, 3]. PCL mainly affects patients in the 5^th^ and 6^th^ decade of life with a male preference [4-6]. Some pediatric cases have been reported in literature [7]. Our patient was 30-year-old. PCL may arise in any part of the body but preferentially involve the extremities, particularly hair bearing areas [4-6]. Localization to the trunk as seen in our case is of a rare occurrence [8-10]. Clinically, patients typically present with a slow-growing solitary nodule with an indolent growth pattern [10, 11]. Overlying skin can be normal or erythematous. The size range is often between 0.3-5cm [11]. Pain and sudden accelerated growth are the most warning signs for which patients seek medical attention. Pruritis, ulceration and bleeding were reported too.

As clinical presentation may be non-specific, the diagnosis is on the basis of the histologic findings and immunohistochemical examination. It is usually made upon a skin biopsy. However, benign findings should not exclude the possibility of a cutaneous leiomyosarcoma [12]. In our report, the diagnosis of leiomyoma was first rendered and surgical removal was the surest way to establish a definite diagnosis. The dermal variant of leiomyosarcoma is rare and the pathologist must be very careful in assessing depth of invasion. PCL is generally confined to the skin but can exhibit focal and superficial extension into subcutis as seen in our case.

At present, there are no consensuses concerning the optimal treatment of PCL. Although there is evidence that wide local excision with negative lateral margins and depth including the subcutaneous tissue and fascia is the mainstay of treatment, the optimal excision margins for PCL remains controversial. As the recurrence rate and metastatic risk of PCL are strongly related to the adequacy of surgery, a large excision that includes 3-5 cm of uninvolved margins is strongly recommended [13, 14]. Otherwise, evidence from several clinical studies has supported a minimum surgical margin of 1cm [4]. Mohs micrographic surgery is also an attractive option to successfully treat PCL [15]. Although surgery remains the only curative treatment, radiation therapy was reserved to some cases considered to be at higher risk of recurrence. The factors taken into account were tumors larger than 5cm, high mitotic rate, positive surgical margins and subcutaneous extension of the dermal tumor [4, 16, 17]. Other prognostic factor predicting how aggressive the disease course will be were reported too: high mitotic rate of > 20 per 10 high-power fields (HPF), lesions greater than 5cm, tumor necrosis of > 50%, vascular invasion, increased tumor depth with fascial involvement, high stage, increased DNA ploidy, high Ki-67 proliferative index and acral distribution [5, 6].

Although our patient has shown numerous factors of bad prognosis including tumor size, subcutaneous involvement and numerous atypical mitotic figures, he didn’t undergo re-excision to obtain wider margins or receive adjuvant therapy. That may be partly due to the presumed benign course of PCL and no standardized treatment regimens. To date, effectiveness of radiotherapy in this setting has not been determined especially that it has been considered as a causative factor itself. Reports have shown that both radiotherapy and chemotherapy should be reserved for cases of recurrence or metastasis [1, 5]. As the disease can run an uncommon aggressive course as seen in our case and it’s mandatory to define the best therapeutic strategy.

Although local recurrence is common occurring in up to 66.7% of the patients, its potential for distant metastases is negligent (0 - 14.3%). According to the very few cases being referred to the literature, lung was the most common site followed by skin [12, 18]. The majority of patients on surveillance develop metastases within two years from the removal of the primary tumor but a long follow-up is advised to deal with the risk of delayed metastases even 6 years later as seen in our case [6].

Because of its scarcity, there are no series to outline the management of metastatic PCL. Indeed, treatment options in metastatic setting are not dictated by the localization to the skin but by the histological subtype [19]. Classical regimens including doxorubicin, ifosfamide, gemcitabine docetaxel and dacarbazine are used [14]. Although, combination of gemcitabine and docetaxel have shown the best cure rate against metastatic leiomyosarcoma with superior progression-free and overall survival, the high incidence of toxicity was the cause that limited the use of this combination in our palliative setting [20].

## Conclusion

Primary CLM are a diagnostic and therapeutic challenge for clinicians. The present case displayed interesting clinical features of a rare case with an uncommon outcome of CLM and highlights the need for more proper standardized treatments.

## Competing interests

The authors declare no competing interests.
